# Genome-Wide Survey of Cold Stress Regulated Alternative Splicing in *Arabidopsis thaliana* with Tiling Microarray

**DOI:** 10.1371/journal.pone.0066511

**Published:** 2013-06-11

**Authors:** Noam Leviatan, Noam Alkan, Dena Leshkowitz, Robert Fluhr

**Affiliations:** 1 Department of Plant Sciences, Weizmann Institute of Science, Rehovot, Israel; 2 Bioinformatics Unit, Biological Services Department, Weizmann Institute of Science, Rehovot, Israel; Michigan State University, United States of America

## Abstract

Alternative splicing plays a major role in expanding the potential informational content of eukaryotic genomes. It is an important post-transcriptional regulatory mechanism that can increase protein diversity and affect mRNA stability. Alternative splicing is often regulated in a tissue-specific and stress-responsive manner. Cold stress, which adversely affects plant growth and development, regulates the transcription and splicing of plant splicing factors. This can affect the pre-mRNA processing of many genes. To identify cold regulated alternative splicing we applied Affymetrix *Arabidopsis* tiling arrays to survey the transcriptome under cold treatment conditions. A novel algorithm was used for detection of statistically relevant changes in intron expression within a transcript between control and cold growth conditions. A reverse transcription polymerase chain reaction (RT-PCR) analysis of a number of randomly selected genes confirmed the changes in splicing patterns under cold stress predicted by tiling array. Our analysis revealed new types of cold responsive genes. While their expression level remains relatively unchanged under cold stress their splicing pattern shows detectable changes in the relative abundance of isoforms. The majority of cold regulated alternative splicing introduced a premature termination codon (PTC) into the transcripts creating potential targets for degradation by the nonsense mediated mRNA decay (NMD) process. A number of these genes were analyzed in NMD-defective mutants by RT-PCR and shown to evade NMD. This may result in new and truncated proteins with altered functions or dominant negative effects. The results indicate that cold affects both quantitative and qualitative aspects of gene expression.

## Introduction

Plants are sessile organisms and as such are unable to escape from harsh environmental conditions. Instead, plants have evolved a variety of mechanisms to cope with these stresses, including stress-regulated transcriptional responses that ultimately lead to biochemical and physiological changes [1]–[4]. Cold stress is one of the major environmental factors that adversely affects plants' growth and development, and limits crops geographical distribution and yield. Hundreds of cold responsive genes have been identified using transcript profiling techniques, indicating that 10%–45% of *Arabidopsis thaliana* transcripts are regulated by cold stress [1], [4]–[11]. The products of cold responsive genes include functional proteins that directly protect the plant, and regulatory proteins that play a role in signal transduction and gene expression [1], [4]–[7], [9], [11]–[15]. Indeed, the expression and splicing of many serine/arginine-rich (SR) genes, which encode splicing factor proteins that are essential for constitutive and alternative splicing [16], change under cold stress [4], [6], [17], [18]. Since cold stress alters the expression of splicing factors it also affects the splicing of precursor-mRNAs (pre-mRNA) of other genes, which may have an adaptive significance.

Alternative splicing is an important post-transcriptional regulatory mechanism that can increase protein diversity and affect mRNA stability. It generates different mature mRNA sequences from a single pre-mRNA. As a result, different proteins are produced from the same gene through the selection of different exons (and introns), in this way expanding the potential informational content of eukaryotic genomes. Another possible outcome of alternative splicing is the modulation of gene expression by generating different mRNA variants with altered stability, translation efficiency or distribution. A range of processes utilize alternative splicing in plants, including flower development, plant growth, seed quality and many other processes, some of which are related to agricultural concerns [19]–[23]. Additionally, changes in splicing are induced by environmental stimuli [13], [23]–[26], thus alternative splicing could play a role in the response to cold stress or other environmental factors.

Whole-genome tiling arrays (WGA) can be used for gene expression analysis, novel transcript detection and discovery of alternative splicing events. With this technology it is possible to study the dynamics of the transcriptome under various conditions and at different developmental stages. WGAs have been used extensively to monitor alternative splicing, and for epigenomic mapping and expression analysis [11], [27]–[36]. Previous results from our lab showed that direct transcript expression analysis using WGAs is particularly amenable for assessing global intron retention in *Arabidopsis* [37]. Global studies to assess stress induced alternative splicing have used sequencing data. Iida *et al*. [17] applied expressed sequenced tags (ESTs) and cDNA data to investigate general and stress-induced splicing. However, traditional sequencing techniques lack low-abundance transcripts and are biased towards transcript termini. Filichkin *et al*. [38] used high-throughput RNA sequencing (RNA-seq) for genome-wide analysis of general alternative splicing. The results of cold stress-induced alternative splicing were obtained from pooled multiple time points that can confound the interpretation.

Here, we employed WGAs to assess genome-wide cold regulated alternative splicing in *Arabidopsis thaliana*. The effects of cold stress on global gene expression in *Arabidopsis* over time have been the subject of several studies using conventional DNA microarray or WGAs [1], [4]–[6], [8]–[11]. However, none of these studies examined cold dependent alternative splicing. In the present study we used the Affymetrix *Arabidopsis* Tiling 1.0R array to identify changes in splicing that were regulated by cold stress. Our analysis revealed many genes that were not previously reported as cold responsive but whose splicing is regulated by cold. A high proportion of the cold regulated alternatively spliced transcripts contain a premature termination codon (PTC). These are potential targets for degradation via the nonsense mediated decay (NMD) process, which prevents the production of potentially toxic truncated proteins [39]–[46]. In order to determine if the cold regulated transcripts are regulated by NMD we analyzed the splicing pattern of select genes in cold treated NMD-impaired mutants using semi-quantitative reverse transcription polymerase chain reaction (RT-PCR) and quantitative RT-PCR (qRT-PCR). The results show that the presence of potential NMD signatures does not result in down regulation of cold regulated alternative splicing. This suggests that cold regulated splicing resulting in PTC is not necessarily coupled with NMD but is a separate process that might result in new proteins.

## Results

### Mapping Affymetrix probes to the *Arabidopsis* genome

Transcriptome analysis using WGAs was explored for its ability to define splicing events regulated by cold. The method developed for the detection of alternatively spliced introns is based on differences in intron probes expression level between control and cold stress treatments and was applied to analyze changes in alternative splicing in *Arabidopsis* after cold treatment.

WGA analysis was carried out using the Affymetrix *Arabidopsis* Tiling 1.0R Array which is comprised of over 3.2 million non-overlapping perfect-match/mismatch probe pairs. The 25-mer oligonucleotide probes are tiled at an average resolution, which is the distance between the centers of adjacent probes, of 35 bp, leaving a gap of approximately 10 bp between probes. The probes sequences are from the minus DNA strand of each chromosome. Each probe on the tiling array interrogates the presence of a sequence in a labeled double-stranded nucleic acid target through hybridization. After hybridization the intensity of the hybridized labeled target is measured. This intensity correlates with the number of targets that hybridized to a specific probe. A number of studies suggest that using mismatched probes increases the variance and is thus imprecise [47]–[50]; therefore, only perfect-match (PM) probes were included in this study.

The probes were mapped to exons and introns based on the gene structures annotation from The *Arabidopsis* Information Resource (TAIR, http://www.arabidopsis.org/) [51] genome version TAIR9. In case of genes annotated as having multiple transcripts, the isoform with the highest number of exons was used for mapping. Thus, each gene was split into as many different segments as possible. After excluding control probes, intergenic probes, probes that aligned to more than one location in the genome and probes that fall on exon/intron borders; a total of 1,579,896 unique PM probes representing 30,328 genes remained.

### Detection of stress regulated alternative splicing

**Figure 1:**
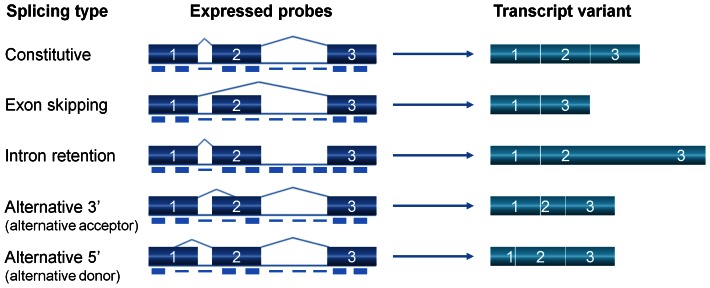
Defining splicing type according to probes expression level. The common forms of alternative splicing represented here are; exon skipping, intron retention, alternative 3' acceptor site and alternative 5' donor site. Boxes joined by lines represent the exons and introns, respectively, of immature transcripts; diagonal lines indicate splicing patterns. Highly expressed probes are depicted as short thick bars while probes with low expression are depicted as short thin bars below the transcript. The splicing variant (right side) is defined as intron retention when all probes in an intron are significantly highly expressed, i.e., have near exon level expression. All other cases of altered intron probes expression are defined as unknown, as the splicing variant can include exon skipping, intron retention or alternative 5' or 3'.

Alternative splicing events were divided into four classes (Figure 1). Exon skipping, which is predominant in mammals [52]–[57], occurs when an exon is sometimes excluded from one transcript and sometimes included in another transcript, this exon is called a cassette exon. An exon can also be lengthened or shortened by alternative donor (5') or acceptor (3') splice sites position. Finally, an intron may not be excised, becoming a part of an exon and resulting in intron retention. A novel algorithm was developed to detect intron retention, which is the most common type of alternative splicing in plants [17], [38], [58]–[62], as well as other changes in splicing (at a resolution of about 35 bp).

To identify splicing that changes under cold stress, total RNA was extracted from the aerial parts of two-week old *Arabidopsis* seedlings after 24 hours of cold (4°C) treatment. Control plants were harvested at the same time to ensure that observed differences would not be due to circadian clock effects on transcripts. The RNA from four biological repeats was converted into double-stranded cDNA and hybridized to *Arabidopsis* Tiling 1.0R Arrays.

Robust multi-array analysis (RMA) was used for background correction and across array normalization [48]. Effects of differences in sample preparation, variance in the microarray reading between each batch of experiments, and other non-biological sources of variations [63] were alleviated by probe-level normalization for all the probes on an array. Normalization between the set of arrays was done by quantile normalization, a method that gives the same distribution of probe intensities to each array in the set [64]. This method may be applied to tiling arrays when all probes are distributed randomly on the array, with respect to their chromosome and location within their chromosome [65]. After background adjustment and normalization the PM values were transformed to logarithmic values (log2) as in this way hybridization values tend to be distributed normally when expressed in log scale [66].

After normalization procedures, probes that might represent stress regulated alternative splicing were detected by comparing the individual expression levels of the intron probes under control and treatment conditions. Next, hybridization values of introns and exons from the whole genome expression database were explored to establish global statistical parameters that can be used to distinguish between intron and exon scores in a transcript. Thus, the expression level of each gene was calculated by averaging the hybridization values of all of the probes that belong to the exons of that specific gene. The mean intensity value of all the probes belonging to the introns of that gene was also calculated. Only genes with low intron expression level but high exon expression level were further analyzed (see Material and Methods). This selection controls for genomic DNA contamination.

To detect differentially expressed introns, each intron's probe intensity from the cold treatment samples was compared to the corresponding probe from the reference (control) samples by using a one-tailed t-test. In this way each probe is assigned a local p value indicating the statistical significance of its differential expression. While the hybridization values are intrinsic to each probe and cannot be compared between different probes of the same intron, the p values of the differential expression of the local intron probes can be compared. Thus, differences in relative hybridization values between neighboring probes, which are due to intrinsic thermodynamic features of each probe or due to technical bias, have no effect.

The p-values of adjacent intron probes were combined using Fisher's combined P method thus providing an overall measure of an intron differential expression, that is, the intron level p-value [67]. To ensure a false discovery rate (FDR) of at most 0.1, the Benjamini-Hochberg (BH) method was applied [68], [69]. Transcripts of genes with intron probes that had an expression level close to the gene’s exons probes expression under treatment condition or control conditions, but not both, and in which the p-value of the t-test was found to be statistically significant were used for defining the gene as undergoing stress regulated alternative splicing. In other words, an intron that is not present in a transcript under one condition but is expressed in a transcript under the other condition is, accordingly, no longer an intron but an exon or part of an exon.

The intron probes, with intensity values that were significantly different (unadjusted p-value <0.05) from those of the matching probes under different conditions, were also used for defining an intron splice type (Figure 1). An intron splice type was defined according to the number of individual probes that differed significantly in their expression. The splice type for an intron that was detected as having significantly higher expression under cold is defined as retained if in addition, all of its composing probes were detected as having a significantly different higher expression than the expression level of the probes in the control group. However, if only part of its composing probes changed it would fall under a different category, and would be defined as "unknown". "Unknown" includes all alternative splicing events such as intron retention, alternative donor (5') or acceptor (3') splice sites or exon skipping. Note that alternative splicing involving short changes, i.e., below 35 bp are beyond the resolution of tiling arrays and would not be detected.

**Figure 2:**
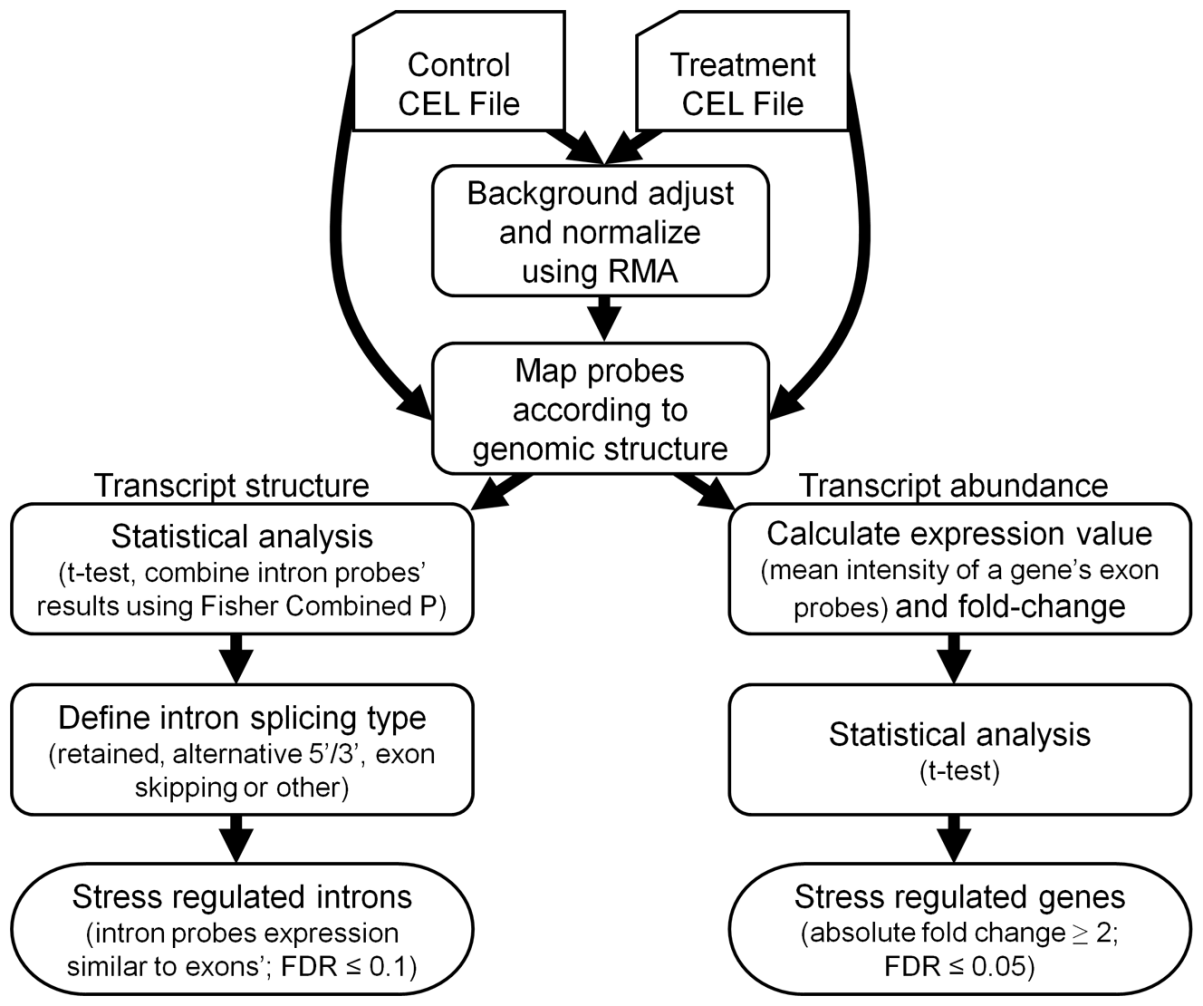
Flowchart of the algorithm used for detecting stress-regulated genes and alternative splicing (see text).

The algorithm was applied to our data and also used to reanalyze tiling microarray data from Matsui *et al.* [11] that had not been used originally for alternative splicing discovery. A summary flowchart for application of the algorithm is shown in Figure 2. RNA-seq data from Filichkin *et al*. [38] were also analyzed using Partek Genomics Suite (Partek GS, Partek, Inc.). The full event list of detected cold-regulated alternative splicing events for 24 h cold treatment data carried out in this work as well as for 2 and 10 h cold data from Matsui *et al.* [11] and cold data from Filichkin *et al*. [38] is available as Table S1. In all, 219 transcripts from 204 genes showed significant differential alternative splicing after 24 h of cold treatment. Of those genes 25% were induced by cold treatment while the rest represent transcripts that have hithertofore not been identified as being modified by cold. A sample list of genes with cold-regulated alternative splicing after 24 h cold is shown in Table 1. The genes were selected randomly for splicing events that include introns that are not completely excised in either cold treatment or control and to be representative of all possible predicted splice types.

**Table 1:** A sample list of detected transcripts with putative stress-regulated alternative splicing.

Gene	Gene cold response (FC)	Intron no.	Type b	Presence c	P-value	EST/cDNA evidence
AT1G11545	-	1	u	cold	6.27E-04	
AT1G24090^d^	-	5	r	ctrl	2.38E-02	
AT1G47530^d^	3.7 a	3	u	cold	2.59E-02	Yes
AT1G55970^d^	-	14	u	cold	3.01E-02	
AT2G39550^d^	-	1	u	ctrl	3.96E-02	
AT2G43160^d^	-	1	u	ctrl	4.00E-03	
AT3G06620^d^	-	4	r	cold	2.63E-02	Yes
AT3G18860	-	14	r	ctrl	3.22E-02	
AT3G22420^d^	-	6	u	ctrl	2.57E-02	Yes
AT3G47630^d^	-	1	u	ctrl	3.95E-02	Yes
AT4G35800^d^	-	13	r	cold	3.99E-02	Yes
AT5G11670	-	15	u	ctrl	4.79E-02	
AT5G24650	-	2	u	cold	1.92E-02	
AT5G26980^d^	-	5	u	cold	2.93E-02	
AT5G56290	-	6	u	ctrl	8.45E-03	

The listed genes were used for validation of predicted cold-regulated alternative splicing events by RT-PCR. All genes, except AT1G47530, were not defined as cold responsive based on showing less than 2 and more than -2 fold change (FDR <0.05).

a Example of cold responsive gene.

b Alternative splicing types are intron retention (r) or unknown (u, possible exon skipping, alternative 5'/3' or intron retention).

c The alternatively spliced intron is not completely excised in either cold treatment or control (ctrl).

d Predictions that were confirmed by RT-PCR.

Comparison between the cold regulated alternative splicing events in response to 2-, 10- or 24-h of cold treatment revealed some common splicing events (Figure S1). The degree of overlap between these treatment groups was larger than expected by chance (2- and 10-h: representation factor (RF)  =  56.4, p<3.42E-70; 24- and 10-h: RF  =  17.5, p<8.83E-08; 24- and 2-h: RF  =  9.3, p<1.22E-05). The tiling data shows highly significant overlap, suggesting that the time differentials are compatible. Similar results were obtained when comparing cold regulated alternative splicing events detected in RNA-seq data of pooled multiple time points from Filichkin *et al*. [38] to those detected by tiling arrays (Figure S2; pooled and 2-h: RF  =  8.0, p<4.24E-09; and 10-h: RF  =  11.2, p<1.26E-09; and 24-h: RF  =  8.7, p<4.97E-06). The overlap within one data source i.e., Matsui *et al*. (Figure S1) is greater than between platforms of tiling and RNA-seq from different sources (Figure S1 and S2). Nonetheless, statistically significant overlaps were obtained in comparisons between these experimental platforms as well.

### Validating the quality of cold treatment

In order to assess the validity of the WGA experiments results, the fold-change of gene expression between treatment and control was calculated. Many genes were detected as cold stress regulated (951; FDR threshold of 0.05). The results were compared to published expression profiles of plants exposed to various stress treatments to test whether the treatment functioned as expected. The comparison includes the results (24h cold treatment) and a series of cold-treatment experiments of 2- or 10-h using WGA probe (Matsui *et al.* [11]). In the later case, only the coding strand was labeled, a technique that was originally used by the authors to differentiate sense or antisense transcripts.

To facilitate this comparison we employed the "Hormonometer" tool [70] (http://genome.weizmann.ac.il/hormonometer/) that applies vector-based correlation analysis to compare the results of an array to an pre-compiled expression index. The expression index was supplemented by a series of abiotic stress experiments from Kilian *et al*. [4]. These include 3, 12 and 24 h of cold responsive experiments as well as heat, salt, drought and osmotic stress.

**Figure 3:**
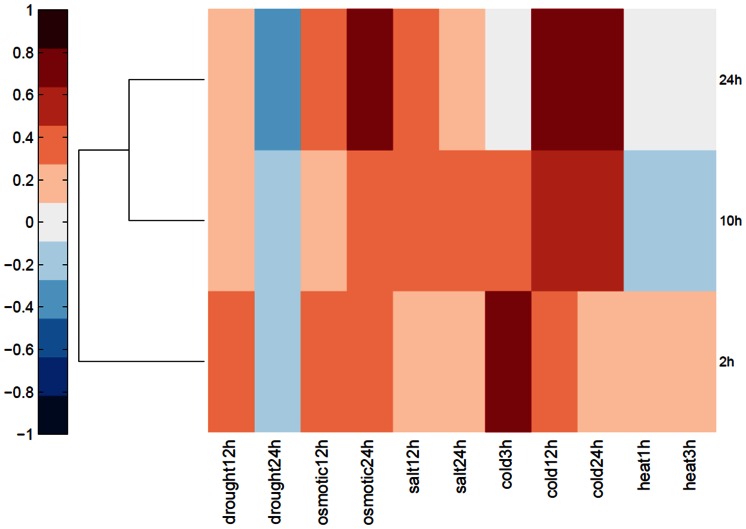
Clustergram representing correlation between transcriptomes from cold treatment conditions and stress treated plants. The transcriptomes of the WGA (2h, 10h, and 24h) experiments are clustered according to their similarity to each stress index, which are shown on the horizontal axis. The indexes for drought, osmotic, salt, cold and heat stress are from Kilian *et al*. [4]. The transcriptomes screened by vector-based analysis are shown on the vertical axis. Correlation values are color-coded from blue (negative correlation) to red (positive correlation). Neutral correlation values are white. The data for 2h and 10h were obtained from Matsui *et al*. [11]

The results of the color-coded vector-based comparison of the cold data are shown in Figure 3 and the actual correlation values appear in Table S2. The results show that the cold treatment at 24 h is highly correlated to previously reported cold treatments of 12 and 24 h but not with the earlier 3 h treatment. The data from Matsui *et al.* [11] are also well correlated with their respective times of cold exposures. Interestingly, there is also a high degree of correlation to osmotic stress which was shown to be mediated by similar pathways [3], [11]. The results validate cold induced expression level data obtained by the WGA probes.

### Validation of genes predicted to undergo stress regulated alternatively splicing

**Figure 4:**
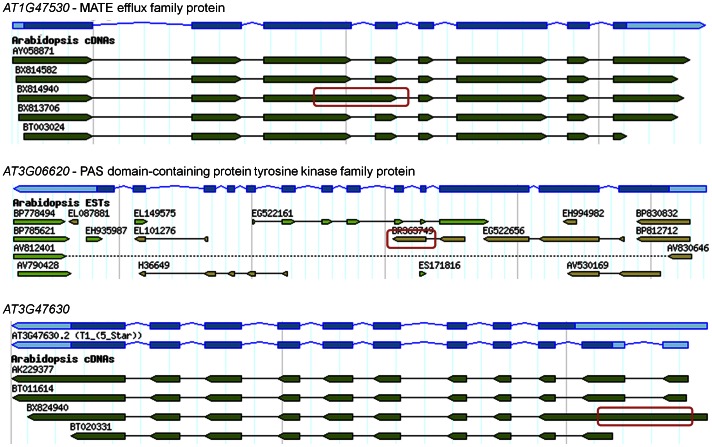
Example of EST or cDNA evidence from TAIR genome browser supporting predicted alternative splicing type. A red rectangle marks the EST or cDNA sequence supporting the predicted alternative splicing of the sample genes *AT1G47530*, *AT3G06620* and *AT3G47630* (Table 1).

Of the 219 transcripts that showed significant differential alternative splicing within 24 h, a sampling of 60 transcripts showed that 30% have supporting EST or cDNA evidence for alternative splicing in TAIR. For example, for the alternative splicing of intron 3 of the gene *AT1G47530*, intron 4 of *AT3G06620*, and the first intron of *AT3G47630* (Figure 4). Note that in the sample list (Table 1) the predicted alternative splicing type for the introns of *AT1G47530* and *AT3G47630* is "unknown", a prediction that represents the possibility of intron retention.

**Figure 5:**
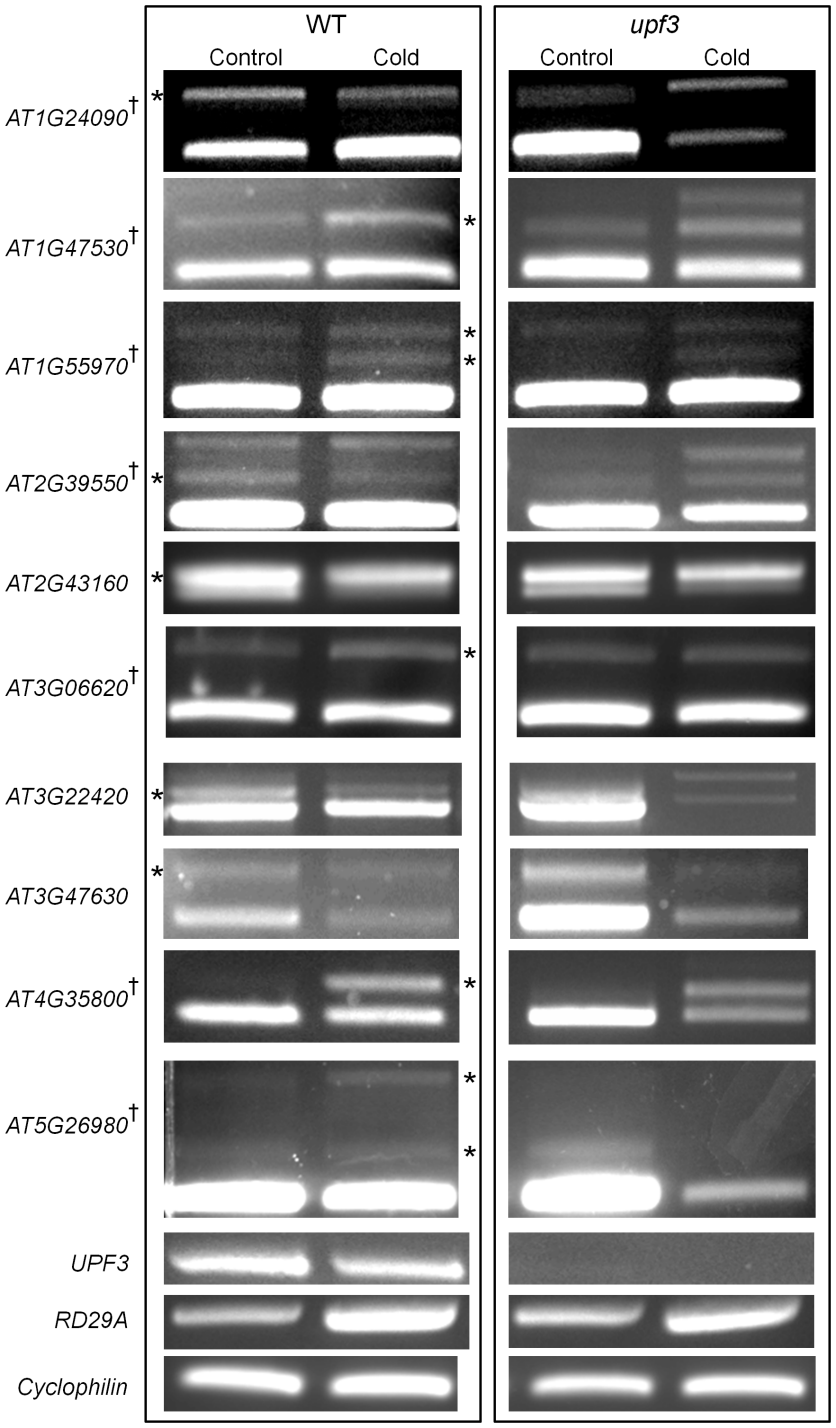
The expression pattern of genes in control and cold-treated plants as examined by RT-PCR. Primers flanking the retained intron were used for amplification. *RD29A* (*AT5G52310*), a gene induced by cold, was used for verification of the effectiveness of the cold-treatment. Amplification of *Cyclophilin* (*AT4G38740*) was used to demonstrate an equal quantity of template in each PCR reaction. Asterisks indicate cold regulated splice variants. Crosses indicate genes with transcripts predicted to trigger NMD (see Figure S3).

To further validate the predictions of cold regulated changes in alternative splicing 15 genes that either had or did not have alternative splicing evidence in EST/cDNA databases and whose transcript level changed, for the most part, by less than two-fold were also analyzed by reverse transcription polymerase chain reaction (RT-PCR). In this analysis the alternatively spliced intron and a juxtaposed intron, not predicted to be alternatively spliced, were included so as to serve as a control for the sensitivity of the tiling array method. The selected alternative splicing events are listed in Table 1 and the RT-PCR products for those that could be verified are shown in Figure 5 (left, asterisks indicate cold regulated splice variants). Thus, of the 15 transcripts, 10 yielded PCR products that contained cold induced splicing differences.

### Alternative splicing and nonsense mediated decay

Many of the cold regulated alternative splicing events (e.g. 94% of putative intron retention events) would result in PTC containing transcripts or transcripts with long 3'-UTRs, which are likely targets for NMD [40], [71], [72]. To examine if NMD plays a role in cold induced transcript change we examined control and *upf3* mutant plants that are compromised for NMD [73]. If the cold-regulated alternatively spliced transcripts are normally degraded by NMD they should accumulate in the *upf3* mutant, where the NMD mechanism is inhibited. The mutant *upf3* was chosen as it has been previously demonstrated that the mutant allele of *upf3*, *upf3-1*, has more severe effects on transcript abundance than other viable NMD impaired mutants, such as *upf1-5*. More transcripts are regulated by *upf3* and most of the transcripts regulated by *upf1* are also regulated by *upf3* [72], [74].

Plants were treated with cold for 24 h and the transcript abundance of wild type and mutant *upf3* were monitored by RT-PCR (Figure 5). Seven of the 10 transcripts are predicted to be targets of NMD due to their structure, and are shown in Figure S3 (crosses, Figure 5). The locations of the putative PTC generated are evenly distributed along the transcripts. In most cases, the abundance and distribution of the putative NMD-targeted transcripts did not increase in the mutants when compared to the wild-type (Figure 5), suggesting that these transcripts are not regulated by NMD. For example, in *AT1G24090* the transcript with the retained intron is more abundant in wild-type control plants compared to cold-treated wild-type plants. This transcript is predicted to be a target of NMD, as the retained intron introduces a PTC, and is expected to accumulate in NMD impaired mutants. However, the abundance of the isoform with the retained intron is decreased in control *upf3* mutants. The alternatively spliced transcript of *AT3G22420* is not expected to have NMD yet its splicing pattern in the cold is completely different between the wild type and mutant plants. In *AT3G47630*, although the transcript with the retained intron in the control has gone up in the mutant, so has the constitutively spliced transcript that does not contain a premature stop codon.

Event-specific qRT-PCR primers could be designed for 3 of the 7 transcripts that were potential NMD targets and were used to examine the ratio of putative NMD-targeted and non-NMD targeted transcripts. As shown in Figure S4, the qRT-PCR analysis did not reveal differences between WT and *upf3* control plants, or between WT and *upf3* cold plants (Figure S4, compare black bars to gray bars). Note the absolute level of the bars in Figure S4 is not comparable to Figure 5 as different primer pairs, each with unique efficiencies, are employed; hence only the ratio need be considered. The results show that cold induced changes in transcripts are not sensitive to NMD.

## Discussion

Most (74%) of the genes detected as undergoing cold-induced alternative splicing include transcripts whose levels remain relatively unchanged under this stress. Of these, about 87% contain changes in the coding region. Thus, despite the lack of change in absolute transcript expression level, the coding capacity can be radically different. These genes have so far been overlooked as regulated by cold stress. Thus, analysis by tilling array reveals new types of stress regulated genes that may play a role in plants response to different environmental conditions. The number of genes with detectable changes in alternative splicing is less than the number of genes that show cold responsiveness based on transcript levels.

With the caveat in mind that absolute levels of different probe are not comparable, it is likely that the alternative splicing events comprise only a relatively small proportion of the genes transcripts. This observation is based on the fact that in no case did the expression level of alternative introns of a particular gene achieve levels similar to those of the expressed exons of that gene. The results of the RT-PCR analysis also suggest that while changes are detectable they are modest. However, isoforms may be tissue specific and the relative change would be masked by the isolation procedures used here.

Similarly, in the detection of changes in SR transcripts only modest changes were evident in the cold [18]. However, such modest changes in transcript architecture can confer biological effects. For example, the *Arabidopsis* resistance (R) gene *resistance to Pseudomonas syringae4* (*RPS4*; *AT5G45250*) confers resistance to *Pseudomonas syringae* only when the regularly and alternatively spliced transcripts are present [75]. Furthermore, the isoform ratios of *RPS4* shift dynamically during the defense response [21]. Likewise, thermal induction of flowering affects the splicing pattern of the floral repressors *FLM* (*AT1G77080*) and *MAF2* (*AT5G65050*) which might be involved in flowering time control [26]. Thus, in some cases such minor changes can show physiological import. It remains to be seen if this is true for the cold responsive genes noted here.

Genes that manifest cold-induced changes in introns present in noncoding regions are also prominent. For example, the gene encoding the largest subunit of RNA polymerase II, *NRPB1* (*AT4G35800*) contains an intron (intron 13) that is located in the 3' untranslated region (UTR) and is retained under cold stress (Figure 5 and Figure S4). This might affect the translational competence of NRPB1 [76]–[79]. Interestingly, *Cyp59* (*AT1G53720*), a multidomain cyclophilin, interacts with the C-terminal domain of the largest subunit of RNA polymerase II as well as with 11 SR proteins. This may indicate that subtle changes in NRPB1 levels may impact on general splicing [80]. Both the splicing pattern of many SR genes [18] and of *NRPB1* are altered under cold-stress, allowing plants to regulate splicing and expression of genes quickly under stress. The cold induced alternative SR transcripts identified by Palusa *et al*. [18] were not detected by our tiling array analysis. This is most likely due to the sensitivity of direct RT-PCR analysis when compared to tiling arrays [18], [72]. As shown here, tiling analysis provides a global view but requires a relatively high degree of expression level suggesting that the cold induced splicing events reported here are an underestimate.

Alternative splicing is a widespread phenomenon that increases transcriptome diversity. It may lead to greater protein variety or may introduce in frame PTCs which could result in transcript degradation by NMD. Coupling of alternative splicing with NMD enables regulation of the abundance of productive transcripts that encodes functional proteins, thus controlling gene expression [81], [82]. Studies of different organisms showed that about 10% of PTC containing transcript are targeted by NMD [81]. Additionally, a recent study found that, in *Arabidopsis*, many splicing factors are affected by NMD [46].

Many of the stress regulated alternative splicing events result in transcripts containing PTCs or having long 3'-UTRs, which are likely targets for NMD. However, our results show that cold regulated alternative splicing patterns of selected genes are mostly unaffected in a mutant defective in NMD. Additionally, in some cases the abundance of PTC containing transcripts actually decreased in the mutants, contrary to the expected effects of NMD inhibition. Furthermore, a recent study by Kalyna *et al*. [72] pointed out that alternative transcripts with retained introns are not removed by NMD, despite having NMD features. Intron retention is the most prevalent type of alternative splicing in plants [17], [38], [58]–[62] and in this work many of the alternative splicing events that are predicted to be of an unknown type are likely intron retention events. For example, the introns of *AT1G47530* and *AT3G47630* are actually retained introns (Figure 4) but are not identified as such by the algorithm. This is because, in analysis of tiling arrays, the type of splicing can be defined as retained only when all intron probes are detected as significantly expressed. Taken together, these data suggest that cold induced alternatively spliced transcripts are not regulated by the NMD mechanism. Thus the observed changes in splicing pattern are probably secondary effects, and are the result of decreased functional transcripts of SR genes in NMD mutants [46].

Stress induced alternative splicing with PTC containing transcripts that are not degraded by NMD may produce truncated proteins. Truncated versions of proteins that lack one or more domains may function as dominant negative regulators [83], [84]. The protein kinase *AT3G06620* gene, for example, undergoes cold induced intron retention (Figure 5 and S4) generating a PTC containing transcript that encodes for a protein truncated before the active domain. The RNase H gene *AT1G24090* alternatively spliced form is more abundant under control conditions (Figure 5) and it also produces a truncated protein lacking the active domain. These truncated proteins may competitively inhibit the full-length proteins under cold or control conditions, respectively. Thus, methodology used here, next generation sequencing technologies or even direct RT-PCR will yield predictive qualitative detection of stress-induced alternative splicing. The ramification of these changes to actual global protein profiles and biological function is a future challenge.

## Materials and Methods

### Plant material, cold treatment and RNA isolation

*Arabidopsis thaliana* (Columbia ecotype) seeds were surface sterilized in 1.5% sodium hypochlorite, 0.005% Triton 100 and 75% ethanol for 1 min, washed in 100% ethanol and allowed to dry. The seeds were germinated and grown in Petri dishes (25 plants per dish) on Murashige and Skoog (MS, Cat. 0222, Duchefa) germination medium, containing 0.8% agar, 3% sucrose and pH 5.8. After stratification at 4°C for 2 days the plants were grown for 2 weeks in a controlled-environment chamber at 22°C under 16h light/8 h dark (long day). For cold treatment, the plants were placed at 4°C for 24h under long day conditions. In order to keep variation between treatment and control to a minimum, cold treatment was started at 11am under light and continued for 24h before the aerial parts of the plants from both groups were harvested. Samples were immediately frozen in liquid nitrogen, and stored at –80°C. Each dish was used as one biological replicate. Total RNA was isolated using RNeasy Plant Mini Kit (Qiagen, http://www.qiagen.com/) according to the manufacturer's instructions. On-column DNase I (Qiagen) digestion was performed during RNA extraction.

*Arabidopsis thaliana upf3-1* (SALK_025175) [85] mutants were grown, treated, and used for total RNA isolation as described above.

### Probe synthesis for tiling array analysis and microarray hybridization

Seven μg per sample of total RNA was used for synthesis of hybridization targets with GeneChip Tiling WT Double-Stranded cDNA Synthesis Kit (Affymetrix) and the GeneChip WT Double-Stranded DNA Terminal Labeling Kit (Affymetrix) according to the manufacturer's instructions. The targets were hybridized to Affymetrix *Arabidopsis* Tiling 1.0R Array, washed on Fluidics Station 450 using Affymetrix protocol FS450_0001 and scanned using Genechip Scanner 3000 7G.

Probe synthesis, labeling, hybridization, washing and scanning were done by Weizmann's Biological Services (http://www.weizmann.ac.il/biological_services/).

Raw data (CEL) files were submitted to NCBI's Gene Expression Omnibus (GEO) [86] (accession number GSE35996).

### Mapping tiling probes to annotated gene models

Affymetrix *Arabidopsis* 1.0R Tiling arrays contain over 3.2 million perfect-match (PM) and 3.2 mismatch (MM) 25-oligonucleotide probes. The PM probe sequences were mapped using BLAT [87] with a threshold of 95% identity to *Arabidopsis* exons and introns, as annotated in TAIR9 genome release [51] (ftp://ftp.arabidopsis.org/Sequences/blast_datasets/TAIR9_blastsets/). In case of genes annotated as having more than one transcript, the exons and introns of the one with the most exons were used, defining the "base" transcript for the analysis. Probes that mapped to multiple locations, probes that fell on exon/intron borders, intergenic probes, control probes and all MM probes were excluded from tiling array analysis. Further analysis was done using the remaining 1,579,896 unique probes.

### Identification of cold-responsive genes

Probe intensity level data were preprocessed using the robust multi-array analysis (RMA) method [48] with background adjustment, quantile normalization [64] and log_2_ transformation. RMA only uses PM probes. Expression values of each gene were defined as the mean intensity of all probes mapped to the exons of the gene. Expression values were only calculated for the 30,328 genes represented by more than three probes. Fold-changes between cold treatment and untreated controls were calculated for all genes by substracting the cold-treated expression value from the control expression value. Genes were identified as stress responsive by a t-test and Benjamini-Hochberg (BH) multiple testing correction to ensure a false discovery rate (FDR) of at most 0.05 [68], [69]. Only genes exhibiting an absolute fold-change of 2 or more were defined as cold responsive.

### Comparing cold-responsive genes from this and other studies

Transcriptome similarities between various stress treatments and the different cold-stress treatments from this study and from Matsui *et al.* [11] were assessed by vector based analysis using a tool based on Hormonometer [70] (http://genome.weizmann.ac.il/hormonometer/). A gene expression index was build for stress treatments comparison in a manner reminiscent of the Hormonometer algorithm [70]: Affymetrix ATH1 microarray CEL files (containing raw data) of stress related experiments were retrieved from GEO. They were imported into Partek GS (Partek, Inc.) [88], RMA normalized [48] and then the fold-change (treatment/control) of the genes and the statistical significance (using one-way ANOVA test) of that change were calculated. An index representing a particular experiment was compiled by selecting, for each of these stress related experiments, the 1,000 genes with the highest absolute fold-change and an unadjusted p-value < 0.05. The index was then compared by vector-based comparison to transcriptome results of the different cold-treatment experiments.

The resulting correlation coefficient is between -1 and 1, with 1 indicating that the direction and intensity of the stress index and the experiment are identical, 0 indicating no correlation, and -1 indicating they are complete opposites.

Data from Kilian *et al*. [4] were downloaded from GEO: cold stress (GES5621), drought stress (GSE5624), heat stress (GSE5628), osmotic stress (GSE5622) and salt stress (GSE5623).

### Detection of genes with cold regulated alternative splicing

Probe intensity level data from this study and from Matsui *et al.* [11] (GEO accession GSE9646) were preprocessed as described above for cold-responsive genes identification. Different processing of introns under stress was assessed for the 20,904 intron containing genes represented by more than eight probes of which at least one probe is located within an intron. The distribution of the expression values of the genes and the distribution of the mean intensity of all probes belonging to the introns of the genes were compared in order to establish parameters for distinguishing between exons and introns. The median and the mode intensities of all probes belonging to genes' exons were calculated. Genes whose expression value is greater than this mean, and whose mean introns expression level is less than the median intensity of the probes of all of the genes, were used for further analysis. That is, genes were selected if most or all of their introns were hardly expressed but their exons expression level was above the calculated threshold.

Testing for differential expression of introns was done by combining the results of comparisons between individual probes as previously described [67]. Briefly, probe level one-sided t-tests were used for calculating p-values. Probes with small p-values which changed in the opposite direction of the one-sided test were assigned the complementary (1-p) value. The p-values for each probe of a single intron were then combined into a single chi-square test statistic using Fisher's method [89]. Note that Fisher's combine P method can only be used for tiling array with completely non-overlapping probes, such as Affymetrix *Arabidopsis* 1.0R Tiling arrays. Another potential source for dependency is the fact that cDNA fragments used as hybridization targets are between 25 and 200 bases and may span multiple probes. In case of retained introns composed of multiple probes, this may lead to correlation between neighboring probes of the same intron. However, it was demonstrated that accounting for this correlation weakened the performance of the combined P method [67] and therefore the potential correlation was ignored here.

The combined p-value was calculated twice, first for one one-sided t-test and then for the other, and the minimal p-value was used as the intron level p-value for the analysis. Introns with an FDR of 10% or less were classified as undergoing cold-regulated alternative splicing. Note that FDR was controlled using BH multiple testing correction method, as above, as an alternative to the computationally more expensive permutation-based estimation.

The plus and minus strand data from Matsui *et al.* [11] were analyzed separately and combined to obtain a list of cold-regulated alternative splicing events

An intron's splice type was defined according to its individual probes responses. If all of the probes of an intron change significantly (unadjusted p-value <0.05), and in the same manner, the intron was defined as retained. In all other cases the type of splicing was defined as "unknown".

RNA-seq data were also used for analysis of cold-regulated alternative splicing of introns. Control and cold stress RNA-seq data [38] were retrieved from NCBI Short Read Archive (SRA009031) The reads were aligned to the *Arabidopsis* genome using TopHat [90], which aligned reads to the genome using the Bowtie [91] algorithm. The resulting files were imported to Partek GS (Partek, Inc.) for quantification and differential expression estimation. Partek quantification estimations were done to the *Arabidopsis* introns using the expectation/maximization algorithm. We restricted the quantification to reads that are completely within the intron. Differential cold versus untreated expressed introns p-values are based on the chi-square distribution.

The resulting lists of cold-regulated alternatively spliced introns were compared by means of Venn diagrams (http://bioinfogp.cnb.csic.es/tools/venny/index.html, Oliveros, J.C. (2007)). The representation factor and statistical significance of the observed overlap was estimated with a hypergeometric test using the web based calculator developed by Jim Lund at the University of Kentucky (http://nemates.org/MA/progs/overlap_stats.html). The representation factor is defined as the number of overlapping introns divided by the expected number of overlapping introns drawn from two independent groups. A representation factor >1 indicates more overlap than expected (enrichment) between the two groups [92].

### RT-PCR analysis

First-strand cDNA was synthesized from 1.5μg total RNA using High-Capacity cDNA Reverse Transcription Kits (Applied Biosystems) with an oligo-dT primer. For control of contamination by genomic DNA, the same reaction was performed without adding reverse transcriptase. PCR amplification was done using primers specific for the 15 selected genes with cold-regulated alternative splicing and for control genes. Primers for the alternatively spliced genes were designed to flank the putative stress regulated intron and a constitutive intron, for simultaneous amplification of both the constitutively spliced and alternatively spliced isoforms. A list of the PCR primers used in this study is available in Table S3. The amplified PCR products were subjected to electrophoresis on 2% agarose gel.

### qRT-PCR analysis

The expression of constitutively spliced and alternatively spliced transcripts of *AT1G47530*, *AT3G06620* and *NRPB1* (*AT4G35800*) was quantified by qRT-PCR analysis using primer designed with Primer Express (Applied Biosystems). The reverse transcription reaction was performed on 1μg of total RNA using the High-Capacity cDNA Reverse Transcription Kits (Applied Biosystems). cDNA samples were diluted 1∶10 to the final template concentration for qRT-PCR. qRT-PCR was performed using StepOnePlus System (Applied Biosystems). PCR amplification was done using 3.4 µl of diluted cDNA template in 10 µl reaction mixture containing 5 µl of SYBR Green amplification kit (Applied Biosystems) and 300nM primers. The cycling conditions were one cycle of denaturation at 94°C for 10 min, followed by 40 three-segment cycles of amplification (94°C for 10s, 60°C for 15s, and 72°C for 20s). The samples were subjected to melting curve analysis. *Cyclophilin* (*AT4G38740*) was used as an internal control for normalization and the relative standard curve was used to calculate the results. Relative expression levels were expressed as transcript change compared to the calibrator sample (WT control) which was set to a value of 1. Primers are listed in Table S3. Each treatment had 3 biological repeats with duplicates for each sample.

## Supporting Information

Figure S1 **Comparison between alternatively spliced introns responsive to 2-, 10- and 24-h of cold treatment**. Venn diagrams showing the overlap between cold-regulated alternatively spliced introns. The degree of overlap between these treatment groups is larger than expected by chance for two independent groups, given that the total number of introns is 121,578 (2- and 10-h: representation factor (RF)  =  56.4, p<3.42E-70; 24- and 10-h: RF  =  17.5, p<8.83E-08; 24- and 2-h: RF  =  9.3, p<1.22E-05). The data for 2h and 10h were obtained from Matsui *et al*.
(TIF)

Figure S2 **Comparison between alternatively spliced introns responsive to cold treatment at different time points**. Venn diagrams showing the overlap between cold-regulated alternatively spliced introns detected in RNA-seq data of pooled time points and in WGA analysis. The degree of overlap between these treatment groups is larger than expected by chance for two independent groups, given that the total number of introns is 121,578 (2h- and pooled: representation factor (RF)  =  8.0, p<4.24E-09; pooled and 10-h: RF  =  11.2, p<1.26E-09; pooled and 24-h: RF  =  8.7, p<4.97E-06). The data for 2h and 10h were obtained from Matsui *et al*. Pooled data were obtained from Filichkin *et al*.
(TIF)

Figure S3 **Models of alternatively spliced transcripts predicted to trigger NMD and their constitutively spliced variant**. The alternative splicing event either introduces a PTC or results in a longer 3' UTR (*AT4G35800*), both features that are predicted to trigger NMD. Gene models based on TAIR9 Genome Browser. Boxes represent exons, diagonal lines represent spliced introns, stop signs represent the stop codon (either PTC or authentic). Light blue coloring represent the UTRs of the constitutively spliced transcript. RT-PCR primer positions are indicated by arrows.
(TIF)

Figure S4 **Relative transcript expression levels in control and cold-treated wild-type and** ***upf3*** **plants**. Relative expression levels (Rel Exp) of the constitutively spliced transcript (grey) and the cold-regulated alternatively spliced transcript (black), predicted to be a target of NMD, of three genes (AT1G47530, AT3G06620 and AT4G35800). The expression level of the constitutively spliced transcript of control wild-type plants was set to 1.0 after normalization relative to *Cyclophilin*. Error bars represent the standard deviations of the means from three replicates.
(TIF)

Table S1 **Cold regulated alternative splicing events for 24 h cold treatment data (this work), 2 and 10 h from Matsui** ***et al.*** **** [**11**] **, and pooled time points from Filichkin** ***et al*** **.** [**38**] **.**
(XLS)

Table S2 **Correlation values for** **Figure 3** **.**
(XLS)

Table S3 **List of primers used for semi-quantitative and quantitative RT-PCR.**
(XLS)
