# Supplementary figures and images for: Genome-Wide Survey of Cold Stress Regulated Alternative Splicing in Arabidopsis thaliana with Tiling Microarray
Source: PLoS One. 2013 Jun 11;8(6):e66511. doi: 10.1371/journal.pone.0066511 (PMC3679080; doi:10.1371/journal.pone.0066511)

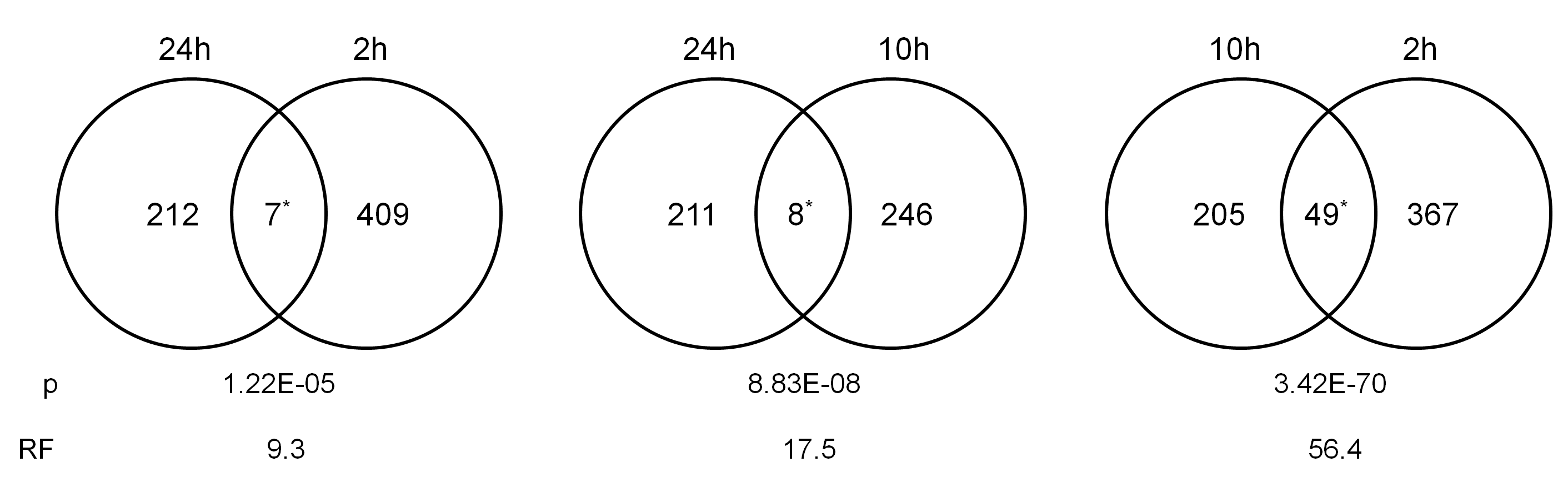

Supplement: Figure S1 — Comparison between alternatively spliced introns responsive to 2-, 10- and 24-h of cold treatment. Venn diagrams showing the overlap between cold-regulated alternatively spliced introns. The degree of overlap between these treatment groups is larger than expected by chance for two independent groups, given that the total number of introns is 121,578 (2- and 10-h: representation factor (RF) = 56.4, p<3.42E-70; 24- and 10-h: RF = 17.5, p<8.83E-08; 24- and 2-h: RF = 9.3, p<1.22E-05). The data for 2h and 10h were obtained from Matsui et al. (TIF) [file pone.0066511.s001.tif]

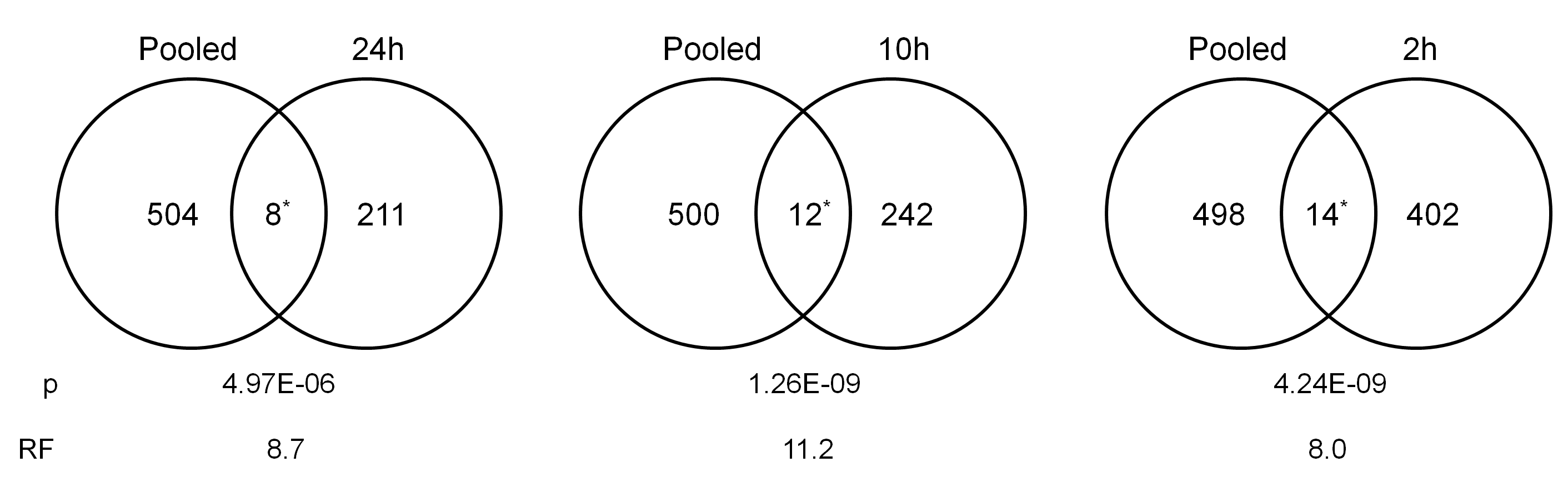

Supplement: Figure S2 — Comparison between alternatively spliced introns responsive to cold treatment at different time points. Venn diagrams showing the overlap between cold-regulated alternatively spliced introns detected in RNA-seq data of pooled time points and in WGA analysis. The degree of overlap between these treatment groups is larger than expected by chance for two independent groups, given that the total number of introns is 121,578 (2h- and pooled: representation factor (RF) = 8.0, p<4.24E-09; pooled and 10-h: RF = 11.2, p<1.26E-09; pooled and 24-h: RF = 8.7, p<4.97E-06). The data for 2h and 10h were obtained from Matsui et al. Pooled data were obtained from Filichkin et al. (TIF) [file pone.0066511.s002.tif]

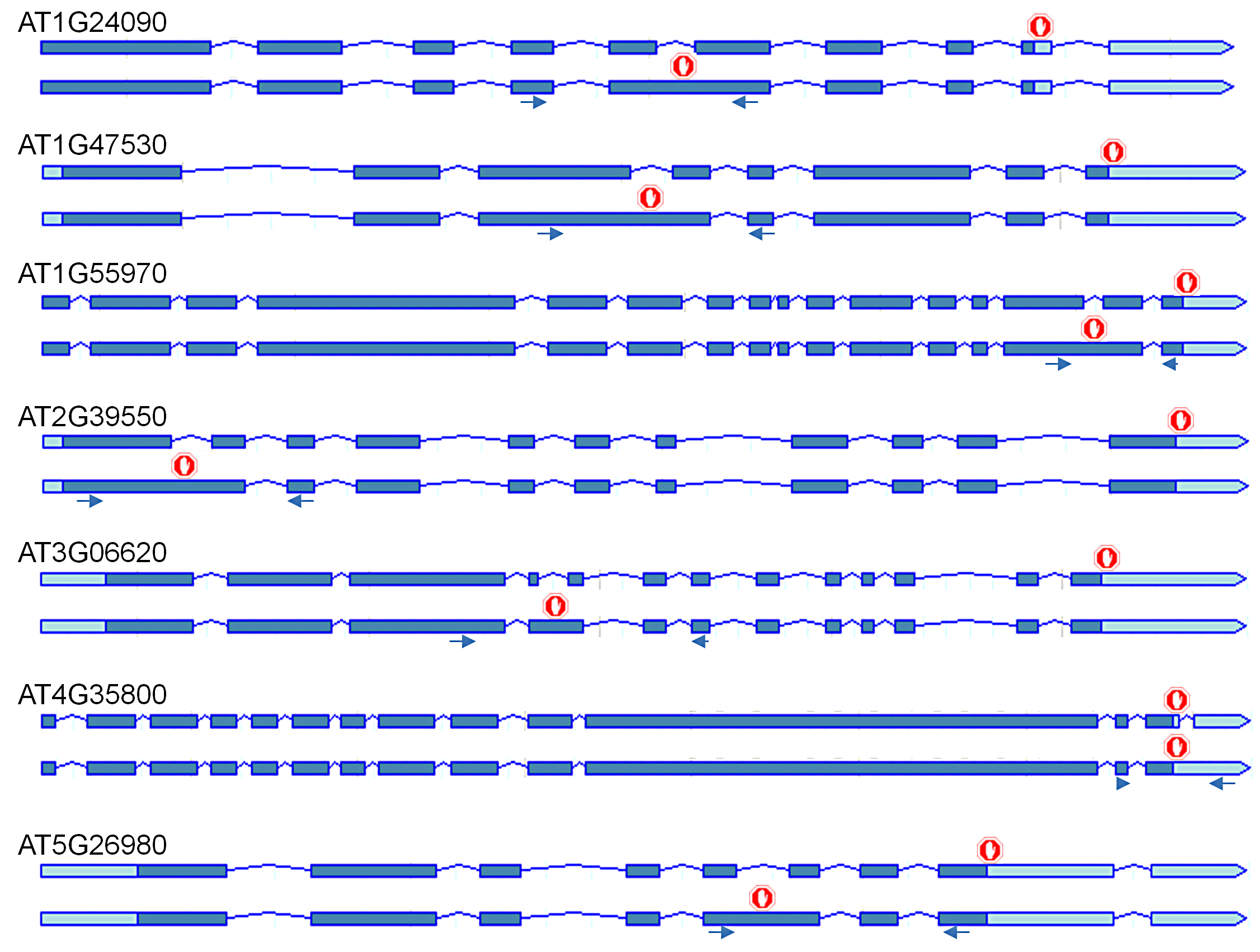

Supplement: Figure S3 — Models of alternatively spliced transcripts predicted to trigger NMD and their constitutively spliced variant. The alternative splicing event either introduces a PTC or results in a longer 3' UTR (AT4G35800), both features that are predicted to trigger NMD. Gene models based on TAIR9 Genome Browser. Boxes represent exons, diagonal lines represent spliced introns, stop signs represent the stop codon (either PTC or authentic). Light blue coloring represent the UTRs of the constitutively spliced transcript. RT-PCR primer positions are indicated by arrows. (TIF) [file pone.0066511.s003.tif]

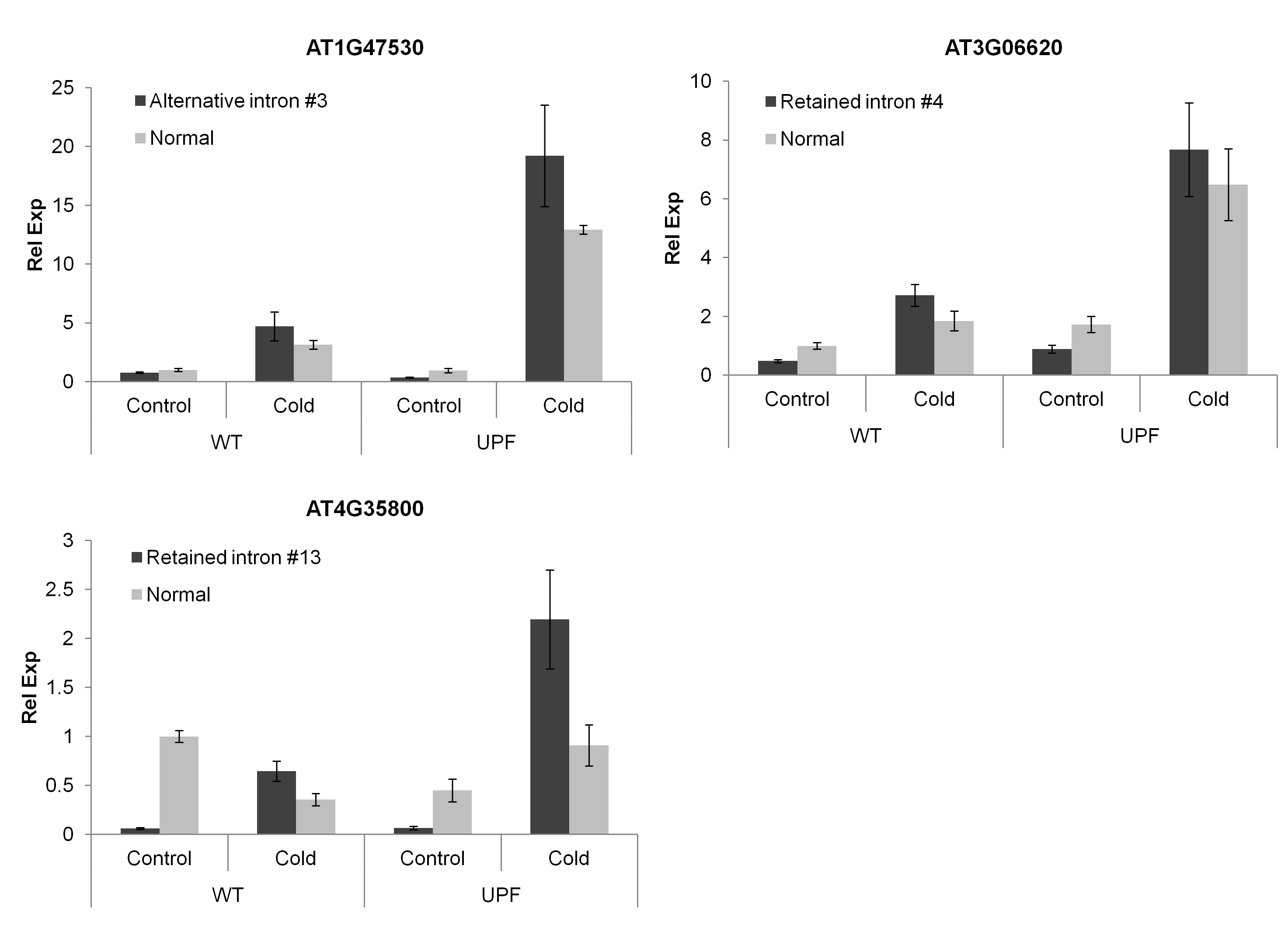

Supplement: Figure S4 — Relative transcript expression levels in control and cold-treated wild-type and upf3 plants. Relative expression levels (Rel Exp) of the constitutively spliced transcript (grey) and the cold-regulated alternatively spliced transcript (black), predicted to be a target of NMD, of three genes (AT1G47530, AT3G06620 and AT4G35800). The expression level of the constitutively spliced transcript of control wild-type plants was set to 1.0 after normalization relative to Cyclophilin. Error bars represent the standard deviations of the means from three replicates. (TIF) [file pone.0066511.s004.tif]
